# Ecological Niche Adaptations Influence Transposable Element Dynamics in Pollinating and Non‐Pollinating Fig Wasps

**DOI:** 10.1002/ece3.71553

**Published:** 2025-06-17

**Authors:** Jing Liu, Yun‐Heng Miao, Hong‐Xia Hou, Da‐Wei Huang, Jin‐Hua Xiao

**Affiliations:** ^1^ Institute of Entomology, College of Life Sciences Nankai University Tianjin China; ^2^ College of Biological Sciences and Engineering Xingtai University Xingtai China

**Keywords:** demographic history, genome evolution, natural selection, phylogenetic analysis, TE landscapes, transposons

## Abstract

This study explores how ecological niches influence the dynamics of transposable elements (TEs) in the genomes of pollinating and non‐pollinating fig wasps (NPFWs), and how these ecological factors shape genome evolution. To examine the protective role of fig fruits for pollinators, we compared TE load and dynamics in six pollinating and five NPFW species from six different *Ficus* species. Phylogenetic analysis was used to assess correlations between genome size, oviposition sites, and TE length. We also analyzed the effects of natural selection and population dynamics on TE accumulation. Significant differences were observed in the total length, number, and types of TEs between pollinators and NPFWs. Phylogenetic analysis indicates that TEs in NPFWs, driven by genome size and oviposition sites, exhibit an expanding state, while pollinators show “dormant” TE landscapes with limited insertions. Despite relaxed selection pressure aimed at prolonging TE retention, pollinators maintain a limited TE abundance, likely due to the contracted population size. Additionally, numerous *cis*‐regulatory modules derived from TEs are located near genes involved in environmental information processing, emphasizing their potential role in adaptation. Our findings highlight the role of ecological niches, represented by oviposition sites, in shaping the TE dynamics of fig wasps. These results provide new insights into how ecological pressures influence genome evolution and adaptation in insects.

## Graphical abstract

The influence of ecological niches on transposable element (TE) dynamics, particularly in mutualistic relationships, remains understudied. This research reveals that non‐pollinating fig wasps (NPFWs) have “aggressive,” expansion‐driven TE landscapes with active insertions, contrasting with the stable, “dormant” TE profiles in pollinators. These findings underscore how habitat stability and oviposition mode shape TE accumulation, highlighting ecological pressures' role in genome evolution and adaptive potential in insects.

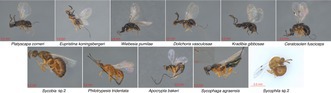

## 1 Introduction

Ecological niches play a critical role in shaping species' adaptation and evolutionary processes. However, how ecological niches influence genome evolution, particularly through transposable elements (TEs) and their temporal and spatial organization in the genome landscape, remains underexplored. TEs contribute significantly to genetic diversity and adaptation, and their activity and genome distribution can reveal not only how ecological pressures influence TE content but also the architecture of genomes. For example, in the invasive ant *Cardiocondyla obscurior*, bursts of TEs (TE islands) associated with olfactory receptor genes facilitate adaptation to new environments (Schrader et al. 2014). Similarly, in the aquatic plant *Nymphoides indica* , more than half of the genome consists of TEs, which may reflect adaptive responses to changing environmental conditions (Yang et al. 2022). In contrast, extreme environments, such as Antarctic ecosystems and enclosed fig fruits, are associated with compressed TE landscapes characterized by low diversity and content, as observed in the midge *Belgica antarctica* (Kelley et al. 2014) and the obligate mutualistic fig wasp *Ceratosolen solmsi* (Xiao et al. 2013). Notably, the specialized ecology of *Drosophila incompta* does not appear to significantly alter either the proportion and diversity of TEs in its genome compared to generalist *Drosophila* species, nor does it establish a distinct TE landscape pattern (Fonseca et al. 2019). These studies suggest that ecological pressures shape TE dynamics in different ways, although results across species have been inconsistent.

In insects, TE content and activity vary widely, leading to dynamic TE landscapes across species (Gilbert et al. 2021). These variations are shaped by a complex balance between TE acquisition, amplification, and loss influenced by both vertical inheritance and horizontal transfer (Walsh et al. 2013; Venner et al. 2017). Extensive studies in arthropods and insects indicate a robust phylogenetic signal and a positive correlation between genome size and TE content, supporting the concept of TEs as hereditary components transferred from parent to offspring (Sessegolo et al. 2016; Petersen et al. 2019; Wu and Lu 2019). However, a study on various *Drosophila* species suggests that at least one‐third of TE families were acquired through horizontal transfer (Bartolome et al. 2009). The replication dynamics of TEs in the genome are intimately linked to their activity, with host‐related factors such as immunity, natural selection, and genetic drift influencing the activity and fate (e.g., loss) of sequences inserted through TE amplification (Szitenberg et al. 2016; Arkhipova 2018; Kofler 2019; Jiang et al. 2024). The interplay of these processes reflects not only intrinsic genetic factors but also the host's ecological and evolutionary context.

**FIGURE 1:**
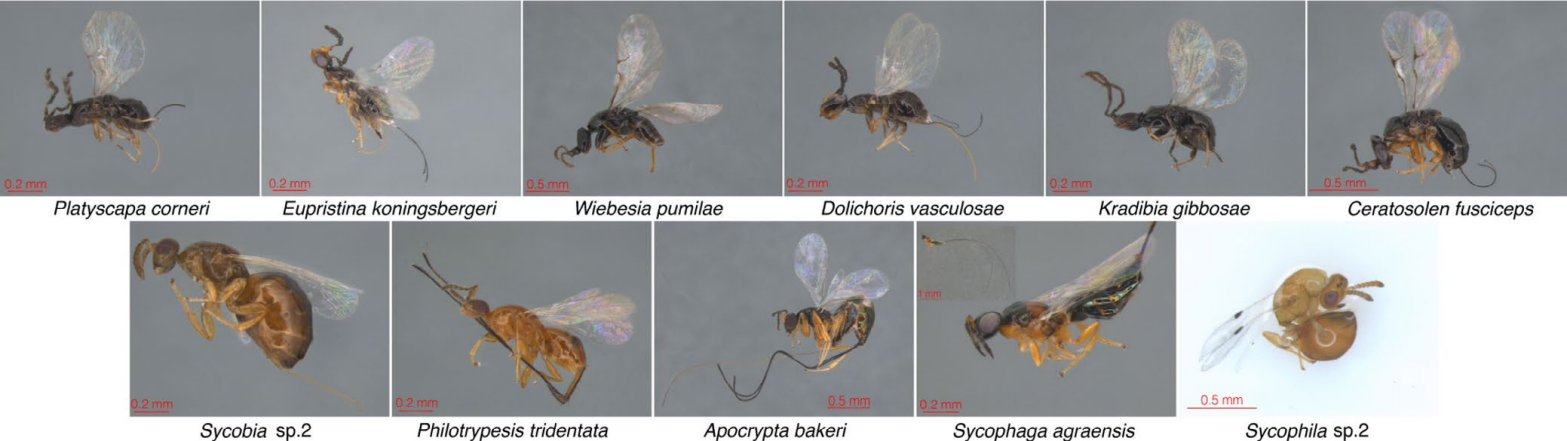
Photograph of pollinators and non‐pollinating fig wasps. The top row represents six pollinator species, while the bottom row shows five non‐pollinating fig wasp (NPFW) species. The red scale bar indicates 0.2–1 mm.

The fig‐fig wasp mutualism provides a unique system to explore the relationship between ecological niche and TE dynamics. Fig wasps are divided into two main groups: pollinating fig wasps (pollinators) and non‐pollinating fig wasps (NPFWs) (Figure 1). As obligate mutualists, pollinators complete their entire life cycle within the syconium and serve as primary colonizers during the female flowering phase. Their morphological adaptations—flattened heads and robust hind legs—facilitate entry through the ostiole, while specialized pollen combs and baskets ensure effective pollination. In contrast, NPFWs exhibit more complex life cycles with broader host interactions. Most NPFWs cannot enter the syconium and have instead evolved elongated ovipositors with interspecific length variation to pierce the fig wall. This ecological divergence extends to niche differentiation: while pollinators colonize syconia synchronously with floral receptivity, NPFWs display distinct temporal and spatial oviposition strategies across ecological guilds (Cook and Rasplus 2003; Ranganathan et al. 2010; Wang et al. 2010). Previous studies have shown distinct genomic adaptations in these groups, with pollinators exhibiting reduced gene families for environmental sensing and detoxification, likely due to their stable and protected habitat inside figs (Xiao et al. 2021). Given these contrasting ecological niches, we hypothesize that: (i) TE landscape divergence. Pollinators, living in a closed, stable environment, will have a “dormant” TE landscape, characterized by the predominance of ancient TE remnants and limited recent TE expansion. In contrast, NPFWs with external oviposition strategies that encounter more variable external environments are expected to exhibit an “aggressive” TE landscape, with ongoing TE insertions and stress‐responsive TE activation. (ii) Circadian system specialization. We further postulate that pollinators' strict oviposition timing—requiring precise synchronization with the brief female phase (typically < 48 h)—has been selected for enhanced circadian clock gene conservation and reduced flexibility in activity rhythms compared to generalist NPFWs.

This study aims to analyze the composition, landscape characteristics, and influencing factors of TEs in the genomes of pollinating and NPFWs. By comparing the TE accumulation and activity between these two groups, we seek to clarify the role of spatial oviposition niches (oviposition sites) in shaping TE dynamics. We also explore how pollinators' precise phenological synchronization relates to the conservation of circadian clock genes and TE regulation mechanisms. Our results will provide new insights into how ecological pressures influence genome evolution and species adaptation, laying the groundwork for future studies on biogeography and conservation in changing environments.

## 2 Materials and Methods

### 2.1 Sample Collection and Genome Assembly

We analyzed six species of pollinating fig wasps (pollinators) and five species of NPFWs, representing all major feeding habits, diverse host fig species, and contrasting oviposition sites. Details of the selected species are presented in Table 1. The voucher specimens have been deposited in the Institute of Entomology, College of Life Sciences, Nankai University, Tianjin, China.

**TABLE 1:** Details of fig wasps and TEs annotations.

Category	Species	Abbreviation	Host fig tree	Collection locality	Collection date	Mating site	Oviposition site	Feeding habit	*r*	*μ*	Genome size (Mb)	TE length (Mb)	No. of TEs	No. of classified TE families	TE content (%)
**Pollinators**	*Platyscapa corneri*	*P. corneri*	*Ficus superba*	Mount Bawang, Hainan	2016/7/11	Inside	Inside	Herbivorous	0.0049	2.43E‐09	304.37	18.23	57,619	56	5.99
	*Eupristina koningsbergeri*	*E. koningsbergeri*	*Ficus benjamina*	Danzhou, Hainan	2016/9/14	Inside	Inside	Herbivorous	0.0051	2.55E‐09	318.36	48.99	113,791	54	15.39
	*Wiebesia pumilae*	*W. pumilae*	*Ficus pumila*	Huangshan, Anhui	2017/4/10	Inside	Inside	Herbivorous	0.0047	2.37E‐09	319.91	25.07	80,474	55	7.84
	*Dolichoris vasculosae*	*D. vasculosae*	*Ficus vasculosa*	Mount Jianfeng, Hainan	2017/4/3	Inside	Inside	Herbivorous	0.0050	2.48E‐09	286.21	8.02	30,895	50	2.80
	*Kradibia gibbosae*	*K. gibbosae*	*Ficus gibbosa*	Xishuangbanna, Yunnan	2016/12/1	Inside	Inside	Herbivorous	0.0052	2.60E‐09	230.30	7.66	42,828	53	3.33
	*Ceratosolen fusciceps*	*C. fusciceps*	*Ficus racemosa*	Xishuangbanna, Yunnan	2016/12/19	Inside	Inside	Herbivorous	0.0053	2.66E‐09	235.18	9.06	57,250	52	3.85
**NPFWs**	*Sycobia* sp.2	*Sycobia* sp.2	*Ficus benjamina*	Nada, Hainan	2017/5/18	Inside/Outside	Outside	Herbivorous	0.0048	2.40E‐09	621.39	235.27	673,913	63	37.86
	*Philotrypesis tridentata*	*P. tridentata*	*Ficus benjamina*	Danzhou, Hainan	2017/5/4	Inside/Outside	Outside	Omnivorous	0.0049	2.45E‐09	398.61	170.35	401,464	65	42.74
	*Apocrypta bakeri*	*A. bakeri*	*Ficus hispida*	Xinglong, Hainan	2017/5/6	Inside	Outside	Omnivorous	0.0051	2.57E‐09	198.59	27.51	146,704	61	13.85
	*Sycophaga agraensis*	*S. agraensis*	*Ficus racemosa*	Xishuangbanna, Yunnan	2017/7/1	Inside	Outside	Carnivorous	0.0048	2.40E‐09	246.14	45.80	114,334	60	18.61
	*Sycophila* sp.2	*Sycophila* sp.2	*Ficus benjamina*	Nada, Hainan	2017/6/10	Inside/Outside	Outside	Omnivorous	0.0043	2.16E‐09	288.59	80.51	252,000	63	27.90

Abbreviations: NPFWs. non‐pollinating fig wasps; *r*, The nucleotide substitution rate per million years per site; *μ*, The neutral mutation rate per generation per site.

The whole bodies of approximately 150 female fig wasps were pooled for DNA extraction for each species. The short‐insert paired‐end library (450 bp) was constructed using the Illumina Kit (San Diego, CA, USA) and sequenced on an Illumina HiSeq 2500 platform, generating ~50× coverage. The long‐insert library (20 kb) was prepared using SMRTbell Express Template Prep Kits (Pacific Biosciences, Menlo Park, CA, USA) and run on a PacBio Sequel system, yielding ~50× coverage.

The PacBio data were corrected with Illumina data by proovread (v2.14.0) (Hackl et al. 2014) and then assembled using SMRTdenovo (v1.0) (https://github.com/ruanjue/smartdenovo). Two polishing strategies were performed with Arrow (Chin et al. 2013) and Pilon (v1.22) (Walker et al. 2014) to correct the assembly with PacBio long reads and Illumina short reads, respectively. The quality and completeness of each genome assembly were assessed by comparing it with BUSCO (v2.0) (Manni et al. 2021) genes, achieving over 95.9% completeness with more than 94.0% single‐copy genes. Duplicated BUSCO ratios ranged from 0.8% to 1.9%.

### 2.2 Transposable Element Annotation

TEs were annotated by constructing *de novo* repeat libraries using RepeatModeler (https://www.repeatmasker.org/RepeatModeler/, v2.0.2) with the “‐LTRStruct” parameter. Libraries were filtered using species‐specific protein annotation files and merged with the “chalcidoidea” TE library, extracted using the Perl script queryRepeatDatabase.pl. from RepeatMasker's util file. We then identified TE families by combining these libraries with RepBase (v20181026) and Dfam (v3.5) databases. For accuracy, the genomes were masked with RepeatMasker (v4.1.2‐p1), using a cutoff parameter of 250. TE annotation results were summarized with the parseRM.pl. (https://github.com/4ureliek/Parsing‐RepeatMasker‐Outputs/) script. Differences in total length, number, types, and genomic content of TEs between pollinators and NPFWs were statistically evaluated using the Mann–Whitney *U* test.

### 2.3 TE Insertion Time Estimation

The insertion times (T) were estimated using the formula *T* = *K*/2*r* (Li et al. 2019), where divergence (*K*) was calculated from Kimura's formula *K* = –300/4 × Ln(1 – D × 4/300), with mutation rates (perc div.) derived for each TE sequence. The nucleotide substitution rate (*r*) was calculated based on 4‐fold degenerate sites from single‐copy orthologs of 17 species, as described by Xiao et al. (2021). Branch lengths were estimated using PhyloFit in PHAST (https://compgen.cshl.edu/phast/, v1.5), and the root‐to‐tip branch length was determined with TreeStat (https://tree.bio.ed.ac.uk/software/treestat/, v1.2). The nucleotide substitution rate was then computed by dividing the root‐to‐tip branch length by the divergence time of *D. pulex* (485 Mya) on the phylogenetic tree (Xiao et al. 2021).

### 2.4 Phylogenetic Analyses

The phylogenetic relationship of 12 fig wasps (including 11 fig wasps in this research and *Ceratosolen solmsi*) was reconstructed using single‐copy orthologous genes, with *Apis mellifera* designated as the outgroup. Single‐copy orthologs were identified using OrthoMCL (v2.0.9) (Li et al. 2003). Protein sequences of each gene were aligned using MAFFT (v7.313) (Katoh and Standley 2013) and poorly aligned regions were removed using Gblocks (v0.91b) (Talavera and Castresana 2007). A maximum likelihood tree was constructed using IQ‐TREE (v1.6.12) (Nguyen et al. 2015) with JTT + F + I + G4 model and 5000 bootstrap replicates.

A phylogenetic tree used for phylogenetic comparative analysis was constructed using TreeViewer (v2.2.0) (Bianchini and Sanchez‐Baracaldo 2024). The phylogenetic signal of TE length, genome size, and oviposition sites was evaluated using Blomberg's K and D values (Fritz and Purvis 2010). A phylogenetic tree‐based Monte Carlo simulation approach was used to evaluate the statistical power of Blomberg's K estimation. Using the fastBM function from the phytools (Revell 2024) package, we generated continuous trait data under Brownian motion models with specified true *K* values (0.9, 1.0, and 1.1). For each simulated dataset, phylogenetic signal strength was assessed via phylosig with significance threshold alpha = 0.05 (testing H_0_: *K* = 0). After 1000 simulation replicates, statistical power was quantified as the proportion of iterations rejecting H_0_.

Relationships between TE length, genome size, and oviposition sites were analyzed using phylogenetic generalized least squares (PGLS) in R with the “APE” (Paradis and Schliep 2019), “phytools” (Revell 2024), and “caper” (Freckleton et al. 2002) packages. Model selection was based on the Akaike Information Criterion corrected (AICc) for small sample sizes. Ancestral trait reconstruction of TE length was performed using the results predicted by the multifactor PGLS model.

### 2.5 Natural Selection Analysis

To detect selection differences between pollinators and NPFWs, we used Codeml from PAML (v4.10.7) (Yang 2007) and the RELAX model in HyPhy (v2.5.57) (Pond et al. 2020). Single‐copy orthologous genes from the 11 species were aligned using MAFFT (v7.520) (Katoh and Standley 2013) and filtered with Gblocks (v0.91b) (Talavera and Castresana 2007) to remove poorly aligned regions. Codeml's free‐ratio model was used to estimate nonsynonymous‐to‐synonymous substitution ratios (dN/dS ratios), and differences between pollinators and NPFWs were compared using the Mann–Whitney *U* test.

Positive selection genes in pollinator lineages were identified using the branch model in Codeml through the likelihood ratio test. Selection intensity analysis was conducted on pollinator branches with the RELAX model, and gene sets under relaxed and intensified selection were collected. To compare the effects of selection pressure on TEs, the lengths of TEs within 1 Kb of these genes were analyzed. To account for differences in TE types near different genes, TE lengths were normalized using consensus sequences.

### 2.6 Demographic History Analysis

Demographic histories were reconstructed for each species using pairwise sequential markovian coalescence (PSMC, v0.6.5) (Li and Durbin 2011) based on whole‐genome resequencing data. Genomic DNA was extracted using the DNeasy blood and tissue kit (QIAGEN GmbH, QIAGEN Strasse 1, 40 724 Hilden, GERMANY) and sequenced with DNBSEQ (DNBSEQ platform developed by BGI) by BGI Genomics Co. Ltd. Reads were aligned to the genome using BWA‐MEM (v0.7.17) (Li and Durbin 2009), and SNP variants were called with BCFtools (v1.8) (Danecek et al. 2021). PSMC was run with specific atomic intervals “4 + 25 × 2 + 4 + 6,” using the neutral mutation rate (*μ*) calculated from the nucleotide substitution rate per million years per site (*r*).

### 2.7 *cis*‐Regulatory Module Prediction

*cis*‐regulatory modules (CRMs) were predicted using SCRMshaw_HD (https://github.com/HalfonLab/SCRMshaw_HD, vHD) (Kazemian and Halfon 2019) with tandem repeats masked by Tandem Repeats Finder (TRF, v4.09) (Benson 1999). Predictions were based on *Drosophila* training data, with window size (‐‐wlen) set to 200 bp with 100 bp shifts (‐‐wshift). To avoid overprediction, the maximum number of hits for top‐scored windows (‐‐thitw) was set to 2000. Finally, the needed hits chosen by Generate_top_N_SCRMhits.pl script were merged with BEDTools (v2.19.1) (Quinlan and Hall 2010) and compared to TE locations to assess overlapping patterns.

### 2.8 Gene Function Enrichment Analysis

For functional enrichment analysis, genes within 1 Kb of classified TEs from major burst peaks and CRMs overlapping with TEs were selected. GO and KEGG enrichment analyses were performed using ClueGO (Bindea et al. 2009) in Cytoscape (v3.9.1) with default parameters. Term‐term interrelation was defined based on shared genes using the kappa score. For better visualization of commonalities, the enrichment networks were merged in the intersection mode, and the nodes were filtered using the parameter Term *p*‐value < 0.05.

## 3 Results

### 3.1 TE Composition of Fig Wasps Across Different Ecological Niches

The total TE length in six pollinating fig wasp (pollinators) species varied between 7.66 and 48.99 Mb, while in five NPFWs species ranged from 27.51 to 235.27 Mb (Table 1). Mann–Whitney *U* test revealed that both the total length (*U* = 28.000, *Z* = 2.373, *p* = 0.017, Figure S1a) and the number (*U* = 30.000, *Z* = 2.739, *p* = 0.004, Figure S1b) of TEs were significantly higher in NPFWs than in pollinators. Pollinators exhibited between 50 and 56 classified TE families, whereas NPFWs ranged from 60 to 65, with significant differences between the two groups (Mann–Whitney *U* = 30.000, *Z* = 2.745, *p* = 0.004, Figure S1c). Among the classified TE families, 46 types were common across all species, while six types were exclusive to NPFWs. The Crypton‐I family was absent in pollinators, except for one detected instance in *Wiebesia pumilae* after parameter adjustments, indicating a trend of TE loss in pollinators.

The genomic content of TEs in pollinators ranged from 2.80% to 15.39%, while in NPFWs, it was significantly higher, ranging from 13.85% to 42.74% (Table 1). The Mann–Whitney *U* test confirmed that these differences in TE content between the two groups were statistically significant (*U* = 29.000, *Z* = 2.556, *p* = 0.009, Figure S1d).

Within the *Ficus benjamina* system, the TE content of three NPFWs (*Sycobia* sp.2, *P. tridentata* , and *Sycophila* sp.2) (mean ± SD: 36.17% ± 7.56%) significantly exceeded that of their obligate pollinator (*E. koningsbergeri*: 15.39%) (one‐sample *t*‐test: *t* = 4.758, df = 2, *p* = 0.041; Table S1), with a 135% elevation in TE load (95% CI: 17.38%–54.95%). This host‐controlled comparison suggests niche‐specific (pollinator vs. NPFWs) TE accumulation independent of host phylogeny.

### 3.2 Ancestral TE Load Reconstruction Indicated Slower Increase in Pollinators

Phylogenetic signal analysis revealed distinct evolutionary patterns across traits. Neither TE length nor genome size showed a significant phylogenetic signal (Blomberg's K for TE length = 0.948, *p* = 0.176; for genome size = 0.863, *p* = 0.357). Phylogenetic tree‐based Monte Carlo simulations revealed limited statistical power (27.4%–29.2%) for detecting Blomberg's K values in the range of 0.9–1.1. However, a significant phylogenetic signal was observed for oviposition sites (D = −5.526, *P*_random_ = 0, *P*_Brownian_ = 0.977).

**FIGURE 2:**
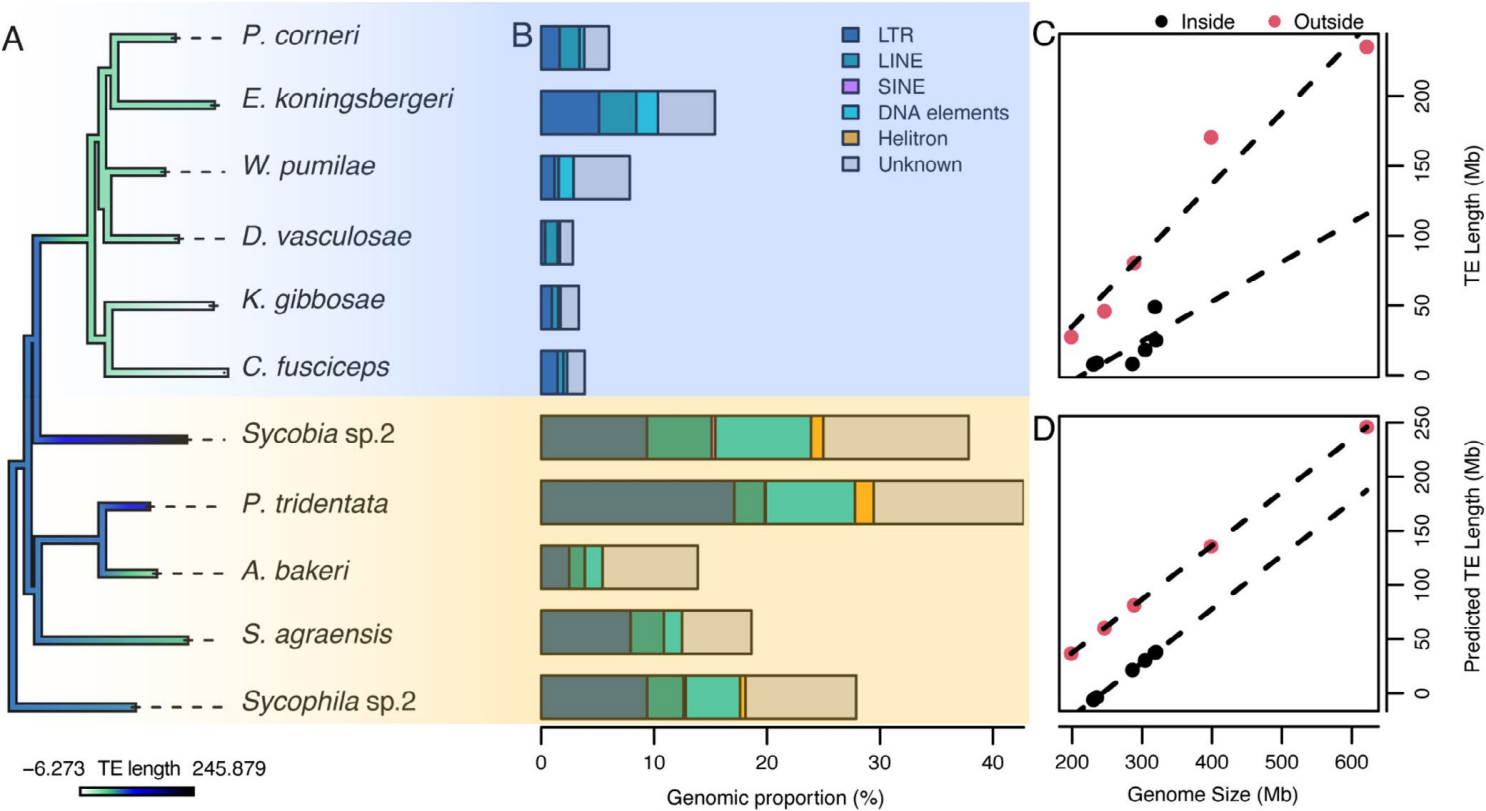
Overview of genomic TE content in fig wasps. Pollinators and non‐pollinating fig wasps (NPFWs) were highlighted with blue and yellow backgrounds, respectively. (A) Phylogeny of the samples in this study, where ancestral state reconstruction reveals a burst in TE length in NPFWs. Branch colors represent TE length estimates. (B) Genomic TE content in fig wasps. LTR: Long terminal repeats; LINE: Long interspersed nuclear elements; SINE: Short interspersed nuclear elements. (C, D) show the relationship between genome size and TE length, as well as the predicted TE length from the PGLS model in relation to oviposition sites. The slope for species with an inside oviposition site (pollinators) is shallower than for species with an outside oviposition site (NPFWs), suggesting that TE length increases more slowly with increasing genome size in pollinators.

Using various PGLS models to explore their relationships, we found that genome size and oviposition sites were independently correlated with TE length (genome size: *λ* = 1.00, *F* = 137.10, *p* < 0.001; oviposition sites: *λ* = 0.00, *F* = 26.06, *p* < 0.01), whereas their interaction was not significant (*λ* = 0.00; *F* = 1.23, *p* = 0.303). The lowest AICc value (97.34) of the multifactor model confirmed the independent effects of genome size and oviposition sites on TE length. The coefficient for genome size (0.495) was notably smaller than that for the “outside” oviposition site (58.594), suggesting that the categorical effect of the “outside” mode had a stronger influence on TE length. As shown in the GLS model (Figure 2C), the increase in TE length in the NPFWs group was faster with the increase in genome size compared to pollinators. Ancestral state reconstruction of TE length (Figure 2A,D), based on the PGLS multifactor model, indicated that TE length in pollinators was shorter than in NPFWs.

### 3.3 Active TEs Shape Distinct Landscapes

**FIGURE 3:**
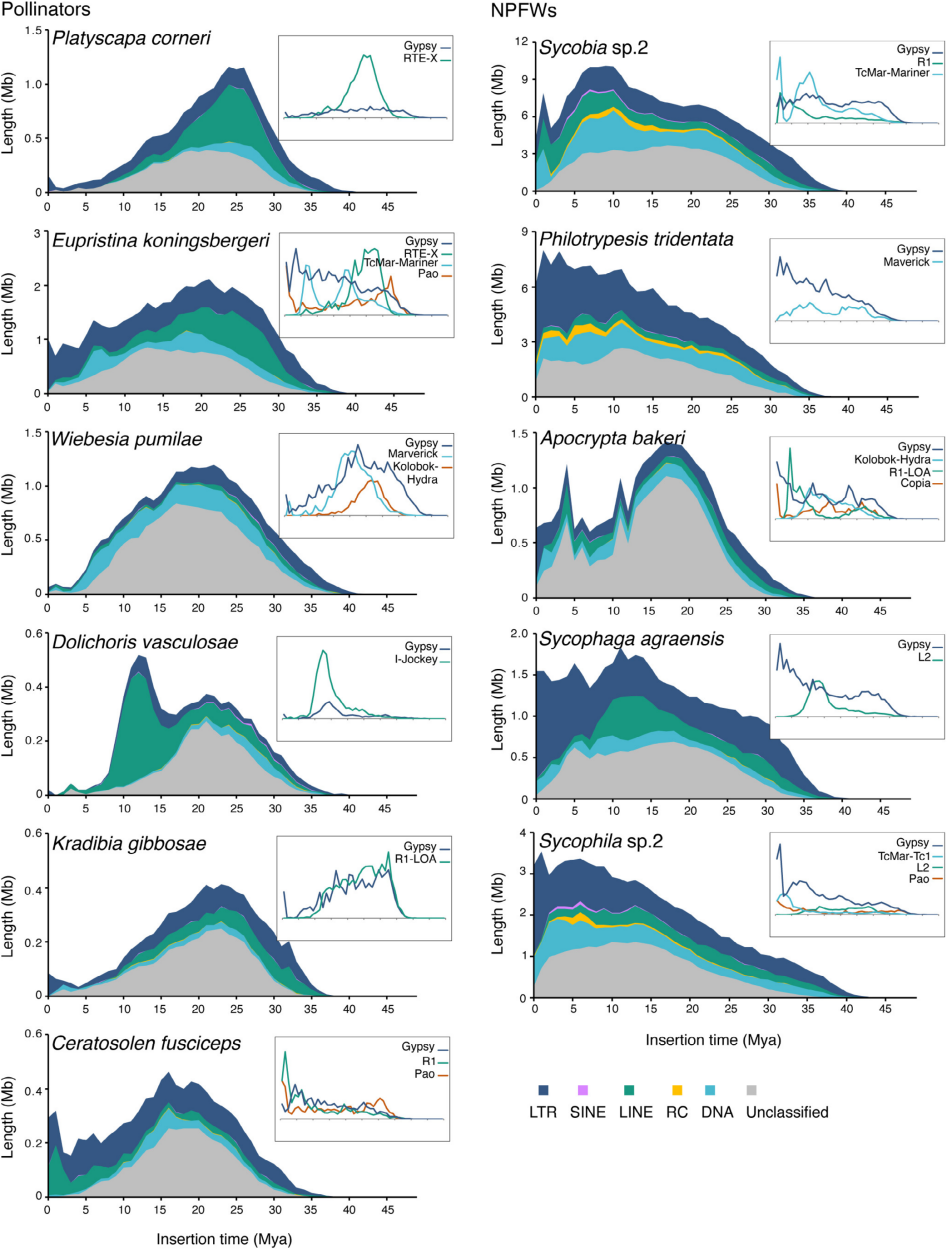
Distribution of TE insertion time in fig wasps. The left panel shows TE landscapes for pollinating fig wasps (pollinators), while the right panel illustrates TE landscapes for non‐pollinating fig wasps (NPFWs). Peaks indicate instances of TE insertion bursts in the genome. Pollinator burst peaks represent ancient, “dormant” states, whereas NPFWs exhibit recent, “aggressive” peaks. Each species' plot includes an *x*‐axis denoting insertion time in million years ago (Mya), and a *y*‐axis showing total TE lengths in million base pairs (Mb). The inset in the upper right corner features a distribution map of selected TE families that make up over 50% of the classified TEs in each species. DNA, DNA transposons; LINE, Long interspersed nuclear elements; LTR, Long terminal repeats; RC, Rolling‐circle transposons; SINE, Short interspersed nuclear elements.

Distinct temporal patterns emerged between pollinators and NPFWs (Figure 3, outside panel). Pollinators exhibited ancient “dormant” peaks (median 77.91% of total TE length from 10 to 30 Mya), reflecting early TEs invasion and amplification followed by suppression and decay, with limited recent TE insertions. In contrast, NPFWs displayed recent “aggressive” peaks, with 17.68% of TE load originating from the last 5 million years (vs. 8.18% in pollinators), highlighting a periodically recurrent influx of active TE insertions.

The dominant TE types varied among species, yet a consistent pattern emerged where 2–4 TE families collectively accounted for over 50% of classified TEs in all species (Table S2). For example, *P. corneri* exhibited a high abundance of the RTE‐X family, with a total length of 4.97 Mb, accounting for 42.7% of classified TEs, while *D. vasculosae* and *K. gibbosae* showed dominance of the I‐Jockey and R1‐LOA families, respectively. Notably, in all NPFWs and several pollinators, the Gypsy family consistently accounted for the highest content, suggesting that bursts of specific TE types have shaped each species' TE landscape (Figure 3, inside panel). Furthermore, landscapes of Gypsy family members in NPFWs (Figure S2) showed that a few members have undergone recent bursts (Figure S2 outside panels), while the multiple bursts of individual members over time indicated sustained genomic activity (Figure S2 inside panels).

### 3.4 Influence of Natural Selection on TEs Accumulation in Pollinators

**FIGURE 4:**
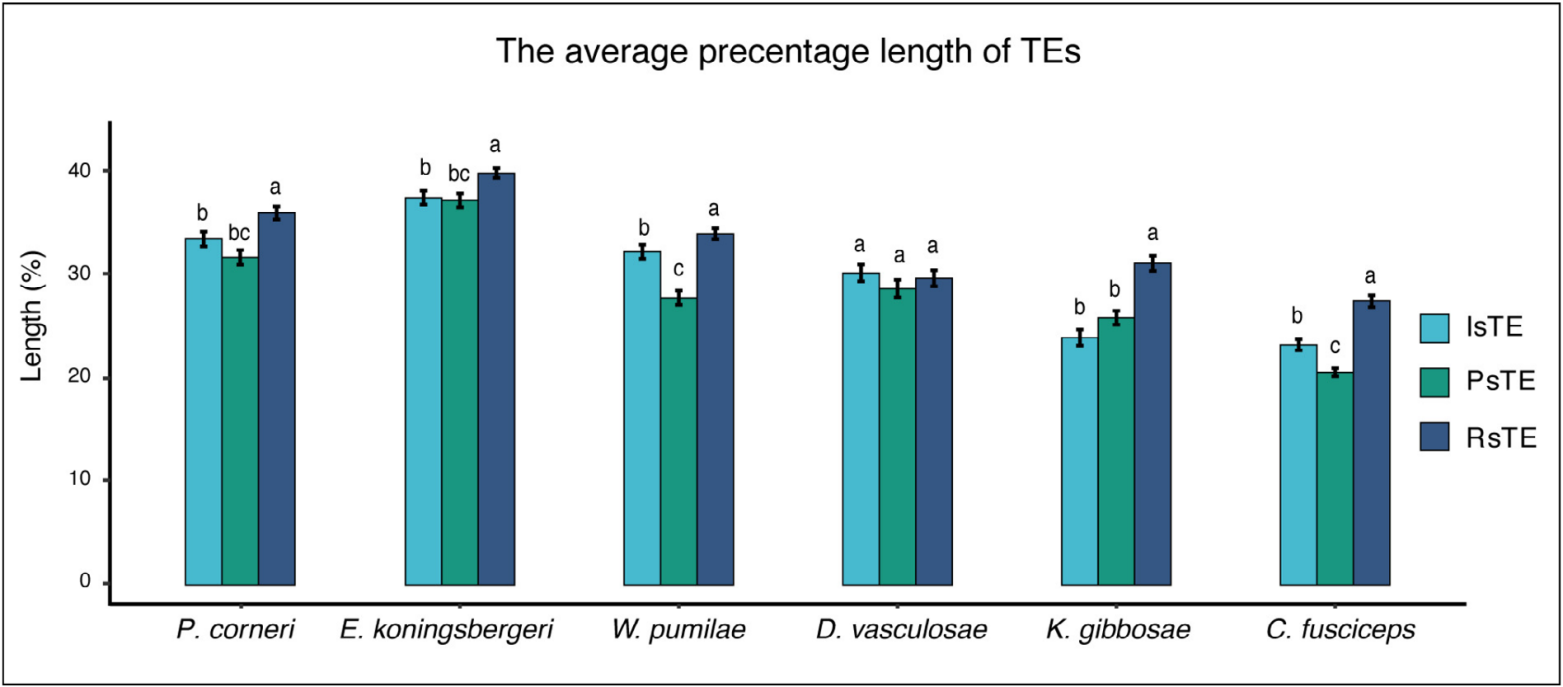
Length distribution of TEs proximal to genes under varying selection pressures in pollinators. Comparisons of TE length near genes under different selection pressures (intensified positive and relaxed) were conducted among pollinators. TE lengths were normalized against their consensus sequence lengths. IsTE represents the percentage length of TEs near genes under intensified selection, PsTE denotes TEs near positively selected genes, and RsTE indicates TEs near genes under relaxed selection. ANOVA followed by Tukey's Honest Significant Difference tests revealed significant differences among the groups. TEs near genes under relaxed selection were generally longer, except in *D. vasculosae*. Different lowercase letters above the boxes denoted significant differences by Tukey's HSD test.

Negative selection was the dominant evolutionary force shaping TE‐related genes in both pollinators and NPFWs (Figure S3). However, nonsynonymous‐to‐synonymous substitution ratios (dN/dS) were significantly higher in pollinators than in NPFWs (ANOVA, *F* = 30.365, *p* < 0.001) (Figure S3). Subsequently, gene sets under relaxed selection (1129), intensified selection (512), and positive selection (642) were identified from 4238 single‐copy homologous genes. The lengths of TEs within 1Kb proximity to these gene sets were compared. Multiple comparisons following ANOVA (Figure 4) revealed that except for *D. vasculosae*, TEs near genes under relaxed selection exhibited significantly longer lengths compared to those near genes under intensified or positive selection. Generally, TEs near positively selected genes had the shortest length, with the exception of *K. gibbosae*.

### 3.5 Historical Population Dynamics of Fig Wasps

**FIGURE 5:**
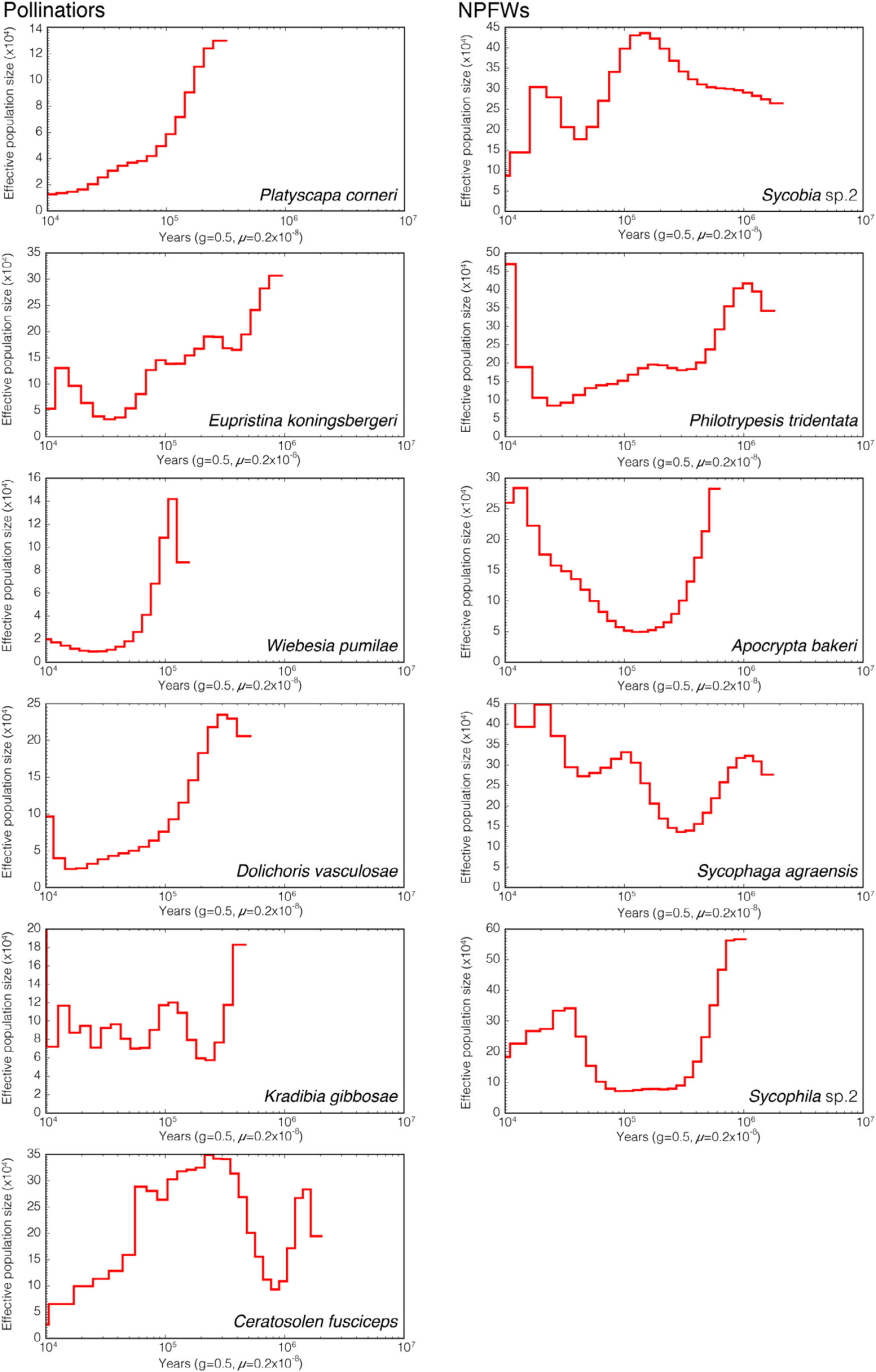
Effective population size dynamics in fig wasps based on pairwise sequential markovian coalescent (PSMC) analysis. The PSMC method estimated changes in effective population sizes (*Ne*) from approximately 2 million years ago (Mya) until 10 thousand years ago (Kya) across all 11 fig wasp species. The left panel shows the *Ne* dynamics of pollinators, and the right panel illustrates the *Ne* dynamics of non‐pollinating fig wasps (NPFWs). Pollinators display a general trend of smaller and declining *Ne*, whereas NPFWs exhibit larger *Ne* values and evidence of expansion. Time was scaled assuming a generation time of 0.5 years and a substitution rate of 0.25 × 10^−8^ per generation.

Effective population size (*Ne*) trajectories revealed distinct evolutionary histories between pollinators and NPFWs over the past 1 million years (Mya) (Figure 5). Pollinators have generally exhibited a decline in *Ne*, with two distinct patterns: a direct decline, as seen in *P. corneri*, and a fluctuating decline, as in *W. pumilae*. In contrast, the *Ne* trajectories of NPFWs were more complex. Some species, such as *P. tridentata* , *A. bakeri* , and *S. agraensis*, showed increases amidst fluctuations, while others, like *Sycobia* sp.2 and *Sycophila* sp.2, displayed fluctuating declines. Recent relative *Ne* values for pollinators ranged from 1.306 × 10^4^ to 9.626 × 10^4^, significantly lower than those for NPFWs, which ranged from 8.717 × 10^4^ to 46.919 × 10^4^ (Mann–Whitney *U* = 29.000, *Z* = 2.556, *p* = 0.009).

### 3.6 Functional Analysis of Genes Near Burst TEs in Pollinators

Temporal patterns of TE burst varied among pollinators, with peak activities at 24–26 Mya (*P. corneri*), 20–21 Mya (*E. koningsbergeri*), 20–22 Mya (*W. pumilae*), 11–12 Mya (*D. vasculosae*), 20–21 Mya (*K. gibbosae*), and 15–16 Mya ( *C. fusciceps* ). Genes adjacent to TE insertion hotspots exhibited significant functional enrichment across pollinator species. Gene Ontology (GO) enrichment analysis revealed significant enrichment in pathways related to ion transport, signal transduction, etc. Similarly, the Kyoto Encyclopedia of Genes and Genomes (KEGG) enrichment analysis indicated significant enrichment in pathways associated with environmental information processing, such as circadian entrainment (term ID: 04713) (Figure S4).

Notably, among the genes enriched in the circadian entrainment pathway, the NOS1 (Nitric Oxide Synthase 1) gene was shared by 5 out of 6 pollinator species. Domain architecture analysis of NOS1 (Table S4) revealed striking differences between pollinators and NPFWs. In pollinators, the NOS1 domains were highly conserved: Domain 1 consistently belonged to the NOS_oxygenase superfamily (cl00254 or cd00795), Domain 2 (FNR_like, flavin‐binding) was retained in all species except for *E. koningsbergeri*, and Domain 3 (electron transfer) featured either CysJ (cl43121) or Flavodoxin_1 (pfam00258). In contrast, NPFWs exhibited frequent domain loss or divergence.

### 3.7 The Evolutionary Relationship Between TEs and *Cis*‐Regulatory Modules

**FIGURE 6:**
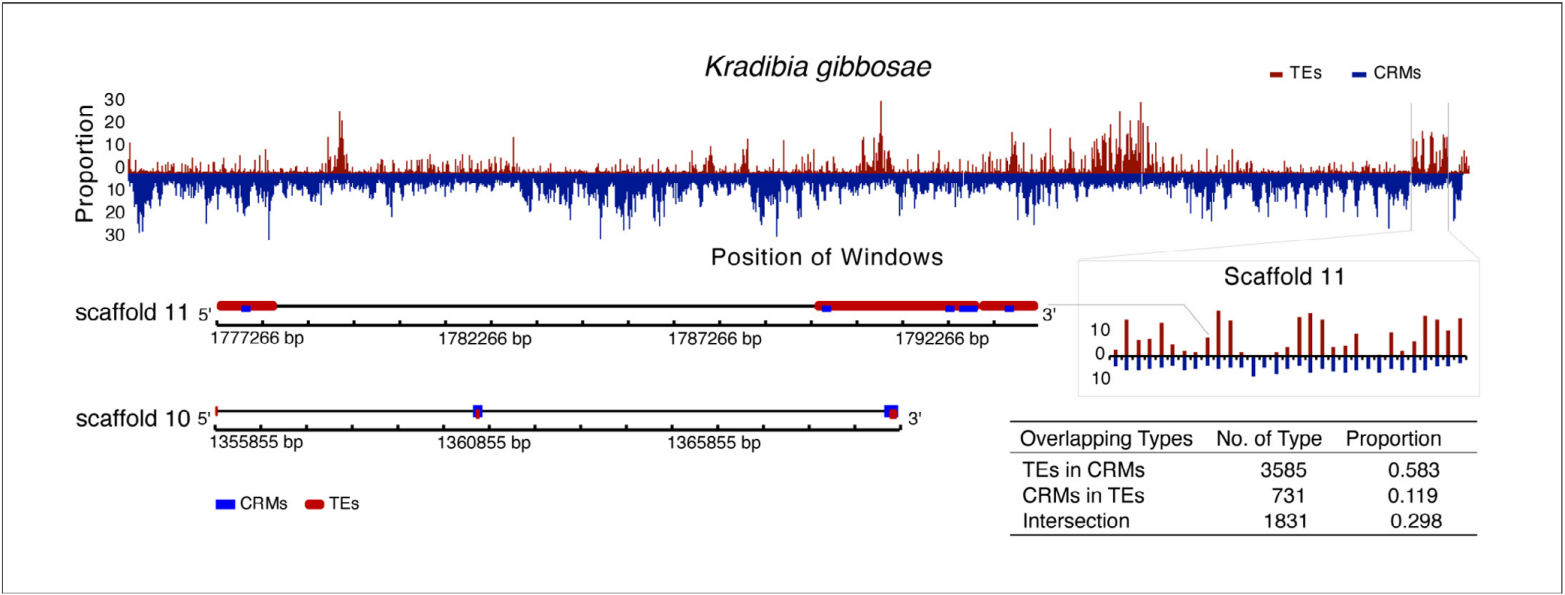
Distribution and overlap patterns between TEs and *cis*‐regulatory modules (CRMs) in *K. gibbosae*. The histogram illustrates the genome‐wide distribution of TEs and CRMs, highlighting areas of overlap. Specific overlapping configurations are shown in partial sequences on scaffolds 10 and 11 with statistical details summarized in the inset table. CRMs are noted for their regulatory potential, and their intersections with TEs suggest potential functional interactions within these genomic regions.

Substantial overlap was observed between TEs and CRMs, with 5.4%–14.1% of TEs intersecting CRMs across pollinator species (Table S3). *Kradibia gibbosae* exhibited the highest proportion of overlap, and its genome‐wide distribution revealed a reciprocal density pattern: regions with high CRM density tended to have fewer TEs, and vice versa. Despite this pattern, substantial overlap was observed in three configurations (Figure 6). The most common configuration was TEs located within CRMs, accounting for nearly 60% of cases, followed by regions where TEs and CRMs intersected. The least prevalent configuration was CRMs within TEs, representing only 11.9%. Furthermore, gene function enrichment analysis of regions near CRMs overlapping with TEs consistently highlighted pathways involved in environmental information processing.

## 4 Discussion

Our analysis of TEs in fig wasps across different ecological niches provides new insights into the factors shaping TE dynamics, including genome size, oviposition sites, selective pressures, and effective population size. The observed differences in TE load and landscape between pollinators and NPFWs highlight the profound influence of ecological constraints and evolutionary histories on genome architecture.

### 4.1 Ecological Niche and TE Dynamics

The lower TE content and family diversity in pollinators, along with the absence of Crypton‐I, may reflect genomic streamlining due to strict vertical transmission and co‐evolution with their host figs. Although Mann–Whitney U tests have limitations when analyzing phylogenetically nonindependent, non‐monophyletic NPFW species, our host‐controlled comparison within the *F. benjamina* system effectively minimized phylogenetic bias while strongly supporting the “niche effect” hypothesis. Despite current sample size constraints, this case study offers compelling preliminary evidence that TE divergence can arise rapidly within shared host environments. These findings are consistent with patterns observed in other host‐specialized insect systems, where niche‐specific selection pressures appear to supersede phylogenetic history (Jousselin et al. 2013).

Our phylogenetic analysis revealed intriguing evolutionary patterns in fig wasp traits. While neither TE length nor genome size exhibited a significant phylogenetic signal, this pattern requires careful interpretation. Monte Carlo simulations confirmed our analysis had limited power to detect moderate phylogenetic signals, suggesting these results may reflect either genuine evolutionary lability or methodological constraints inherent to small clade studies. Despite this limitation, our PGLS models suggest that TE length is still influenced by genome size in a way that aligns with phylogeny. This finding supports the notion that larger genomes tend to accommodate more TEs (Elliott and Gregory 2015). However, the most significant predictor of TE length was the oviposition sites, with external oviposition (“outside” mode) linked to larger TEs. This suggests that exposure to external environmental pressures may facilitate or maintain higher TE activity (Roquis et al. 2021), supporting the hypothesis that TE dynamics are shaped by the complexity and stability of ecological niches.

Pollinators, living in a stable and closed environment within fig fruits, may be subject to stronger suppression mechanisms due to the limited need for rapid adaptation (Bourque et al. 2018), and exhibit a “dormant” TE landscape, characterized by ancient TE insertions that have likely decayed over time. In contrast, NPFWs face diverse parasitic strategies and complex feeding habits, with some oligophagous species interacting with more than one host fig species, necessitating heightened abilities for host and food source detection (Borges 2015). Moreover, the extended duration that NPFWs spend on the external surfaces of syconia for mating and oviposition exposes them to predation and disease risks, potentially driving a need for enhanced defense mechanisms (Ranganathan et al. 2010). Additionally, gallers such as pollinators are proovigenic—emerging with their full complement of mature eggs—while parasitoids are synovigenic, maturing eggs gradully over time (Borges 2015). This difference may be related to the extent to which their eggs are exposed to external environment influences. In addition to the factors discussed above, other ecological traits—particularly the extended adult lifespan and foraging requirements of NPFWs—may further contribute to observed TE dynamics. It has been reported that TEs become active under stress conditions (Horvath et al. 2017), and the active state is a prerequisite for the burst of TEs (Belyayev 2014). This is consistent with the “aggressive” TE landscape, with recent bursts and ongoing TE activity. For instance, the Gypsy family of TEs, which accounts for a large proportion of the total TE load in NPFWs, has undergone multiple bursts, highlighting the active nature of these elements. This indicates that TEs in NPFWs are more likely to be driven by intrinsic TE activity, rather than horizontal transfer.

### 4.2 Selection Pressures and TE Accumulation

Selection pressures, especially purifying selection, often limit TE accumulation by removing deleterious or neutral TEs from the genome (Stritt et al. 2018). Our analysis shows that both groups are predominantly under negative selection, with pollinators exhibiting higher dN/dS ratios, which means more relaxed purifying selection and indeed prolonged retention of TEs within their genomes. This trend indicates that TE accumulation is modulated by the selective context of neighboring genes (Baduel et al. 2019). However, it was apparent that this factor alone did not serve as the explanation for the observed disparity in TE load between the two distinct categories of fig wasps.

### 4.3 Role of Effective Population Size

Effective population size also has significant and multifaceted effects on the accumulation of genetic variation. In smaller populations, the strength of genetic drift is higher, which can obscure the effects of natural selection and allow deleterious mutations to persist (Mathur et al. 2023). However, the smaller *Ne* combined with relaxed purifying selection clearly cannot explain the lower TE load in pollinators. An alternative perspective suggests that when populations contract, deleterious mutations are more likely to be eliminated (Xie et al. 2022). This is more likely the reason for the lower TE load in pollinators.

### 4.4 TEs as Potential Drivers of Adaptation

The concentrated peak of residual TEs in the genomes of pollinator groups over a specific timescale piqued our curiosity. Considering that TE insertions or deletions could emerge as a potent factor impacting the expression of neighboring genes (Hollister and Gaut 2009; Oliver and Greene 2011), we conducted an in‐depth analysis of TE fragments concentrated from approximately 12 to 24 million years ago (Mya) in pollinators. A predominant association was observed between these TEs and genes involved in signal transduction and environmental information processing. Further investigation into the relationship between TEs and CRMs suggests that these residual TEs may function as CRMs, influencing the expression of nearby genes in response to environmental stress. An illustrative example is the role of TEs in circadian entrainment; as climate‐induced changes affect fig phenology, circadian rhythm regulation may help fig wasps adapt to these shifts (Wang et al. 2024). The highly conserved NOS1 gene in pollinators serves as compelling evidence when compared to NPFWs. This supports the idea that TEs, far from being mere genomic “junk,” may serve important regulatory functions that contribute to host adaptation in response to ecological niche pressures.

## 5 Conclusion

This study underscores the significant impact of ecological niches on the accumulation of TEs in fig wasps. The correlation between genome size and TE content reflects a universal genomic scaling law, but in fig wasps, this relationship seemingly functions independently of phylogenetic constraints, an aspect that warrants further investigation. Instead, the variations in TE activity are primarily driven by ecological pressures and niche‐specific factors. These findings reveal how ecological differences influence the complex dynamics of TE accumulation. Future research could refine methods to track TE gains and losses over time, providing deeper insights into TE evolution and its role in host adaptation.

## Author Contributions

**Jing Liu:** data curation (equal), formal analysis (lead), funding acquisition (lead), investigation (equal), methodology (lead), software (equal), visualization (lead), writing – original draft (lead), writing – review and editing (lead). **Yun‐Heng Miao:** formal analysis (supporting), methodology (supporting), software (equal). **Hong‐Xia Hou:** formal analysis (supporting), funding acquisition (supporting), investigation (equal). **Da‐Wei Huang:** funding acquisition (equal), project administration (lead), writing – review and editing (equal). **Jin‐Hua Xiao:** project administration (lead), resources (equal), writing – review and editing (lead).

## Conflicts of Interest

The authors declare no conflicts of interest.

## Supporting information


**Figure S1.** Mann–Whitney *U* Test Results for TE and Genome Metrics in Fig Wasps. The figure presents the statistical comparisons of transposable element (TE) metrics—including TE length (a), TE count (b), TE type (c), and TE content (d)—as well as genome size (e) between fig wasp groups. These comparisons highlight significant differences in genomic TE characteristics between pollinating and non‐pollinating fig wasps.
**Figure S2.** Gypsy landscape in non‐pollinating fig wasp species. This figure illustrates the distribution of Gypsy transposable elements across various non‐pollinating fig wasp species. The outer panels depict Gypsy insertion patterns and recent bursts in each species, highlighting the frequency and timing of new insertions. The inner panels focus on individual Gypsy elements, revealing multiple burst events over time and showcasing the ongoing and sustained activity of these elements within the genomes.
**Figure S3.** Selection analysis of single‐copy orthologous genes in fig wasps. This figure compares the dN/dS ratios of all single‐copy orthologous genes between pollinating fig wasps (pollinators) and non‐pollinating fig wasps (NPFWs). The median dN/dS ratio is significantly higher in pollinators than in NPFWs, indicating a difference in selective pressure between the two groups (ANOVA, *p*‐value < 0.001).
**Figure S4.** GO and KEGG network enrichment analysis of genes near classified TEs at the major peak in pollinators. This figure presents the Gene Ontology (GO) and Kyoto Encyclopedia of Genes and Genomes (KEGG) enrichment analysis for genes located near transposable elements (TEs) at the major insertion peak observed in pollinating fig wasps. Only terms with a *p*‐value < 0.05 were included, as indicated by the filtered nodes in the network.
**Table S1.** One‐sample t‐test for fig wasps in *Ficus benjamina* . This table presents the results of a one‐sample *t*‐test comparing the observed traits (genome size, TEs length, TEs count, and TEs content) of NPFWs (*Sycobia* sp.2, *Philotrypesis tridentata*, and *Sycophila* sp.2) associated with *Ficus benjamina* against its pollinator (*Eupristina koningsbergeri*). TEs Content of NPFWs show mean differences from the pollinator (*p* < 0.05), with Cohen’s *d* effect sizes (*d* < 0.8 indicates small effects).
**Table S2.** Detailed statistics of TEs annotations. This table provides comprehensive statistics on the transposable element (TE) annotations across the analyzed fig wasp species. Data include classified TE families, total TE count, TE lengths, genomic content percentages, and specific family distributions, allowing for detailed comparisons of TE composition and abundance in pollinators versus non‐pollinating fig wasps.
**Table S3.** Information on classified TEs overlapped with predicted CRMs. This table details the overlap between classified transposable elements (TEs) and predicted cis‐regulatory modules (CRMs) across pollinator species. It includes statistics on the number of overlapping instances and percentage overlaps with CRMs, providing insight into the potential regulatory impact of TEs on gene expression related to environmental adaptation.
**Table S4.** Domain Architecture of the NOS1 Gene Across Pollinator and Non‐Pollinator Fig Wasp Species.

## Data Availability

Data are available under NCBI BioProjects PRJNA641212 and PRJNA494992. Currently released files include historical genome assemblies; manuscript‐specific data will be fully accessible upon publication (or to 2027‐12‐31). Accession numbers: SAMN44830401, SAMN44830400, SAMN44830399, SAMN44830398, SAMN44830396, SAMN44830397, SAMN44830395, SAMN44830402, SAMN44830394, SAMN44830393, SAMN44830392.
