# Supplementary figures and images for: Ecological Niche Adaptations Influence Transposable Element Dynamics in Pollinating and Non‐Pollinating Fig Wasps
Source: Ecol Evol. 2025 Jun 17;15(6):e71553. doi: 10.1002/ece3.71553 (PMC12173833; doi:10.1002/ece3.71553)

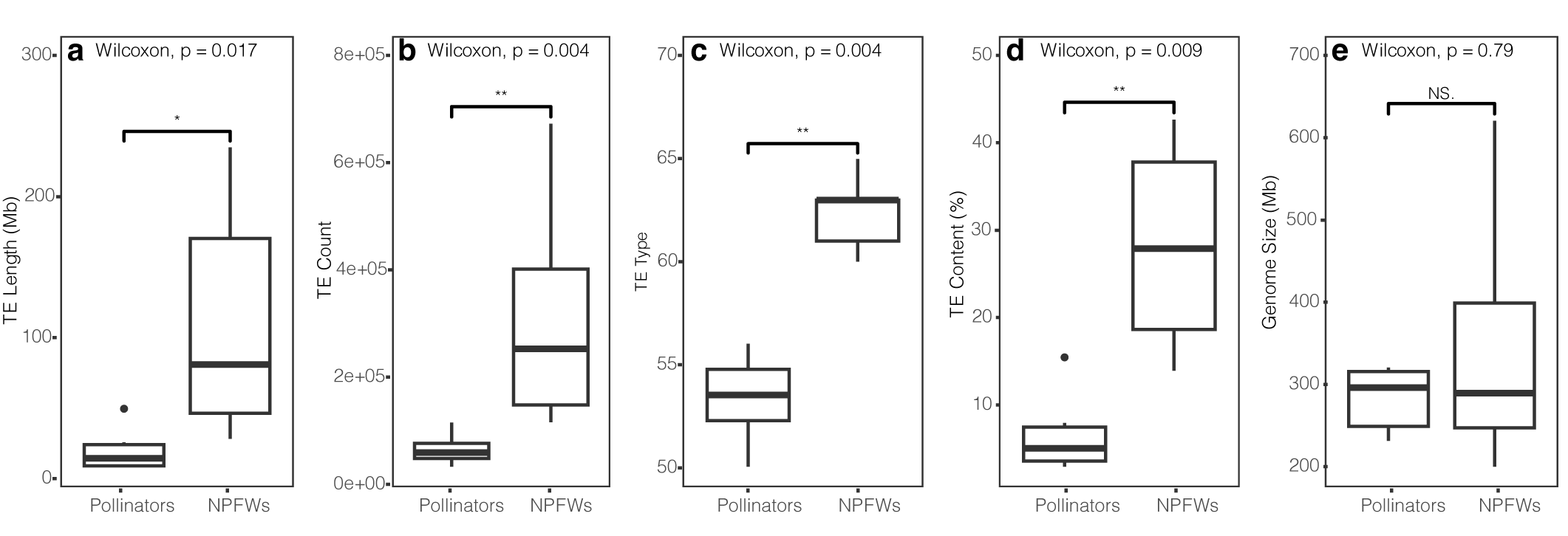

Supplement: Supplementary file 1 — Figure S1. Mann–Whitney U Test Results for TE and Genome Metrics in Fig Wasps. The figure presents the statistical comparisons of transposable element (TE) metrics—including TE length (a), TE count (b), TE type (c), and TE content (d)—as well as genome size (e) between fig wasp groups. These comparisons highlight significant differences in genomic TE characteristics between pollinating and non‐pollinating fig wasps. Figure S2. Gypsy landscape in non‐pollinating fig wasp species. This figure illustrates the distribution of Gypsy transposable elements across various non‐pollinating fig wasp species. The outer panels depict Gypsy insertion patterns and recent bursts in each species, highlighting the frequency and timing of new insertions. The inner panels focus on individual Gypsy elements, revealing multiple burst events over time and showcasing the ongoing and sustained activity of these elements within the genomes. Figure S3. Selection analysis of single‐copy orthologous genes in fig wasps. This figure compares the dN/dS ratios of all single‐copy orthologous genes between pollinating fig wasps (pollinators) and non‐pollinating fig wasps (NPFWs). The median dN/dS ratio is significantly higher in pollinators than in NPFWs, indicating a difference in selective pressure between the two groups (ANOVA, p‐value < 0.001). Figure S4. GO and KEGG network enrichment analysis of genes near classified TEs at the major peak in pollinators. This figure presents the Gene Ontology (GO) and Kyoto Encyclopedia of Genes and Genomes (KEGG) enrichment analysis for genes located near transposable elements (TEs) at the major insertion peak observed in pollinating fig wasps. Only terms with a p‐value < 0.05 were included, as indicated by the filtered nodes in the network. Table S1. One‐sample t‐test for fig wasps in Ficus benjamina . This table presents the results of a one‐sample t‐test comparing the observed traits (genome size, TEs length, TEs count, and TEs content) of NPFWs (Sycobia sp [file ECE3-15-e71553-s001.zip › Figure_S1_SuppInfo.tif]

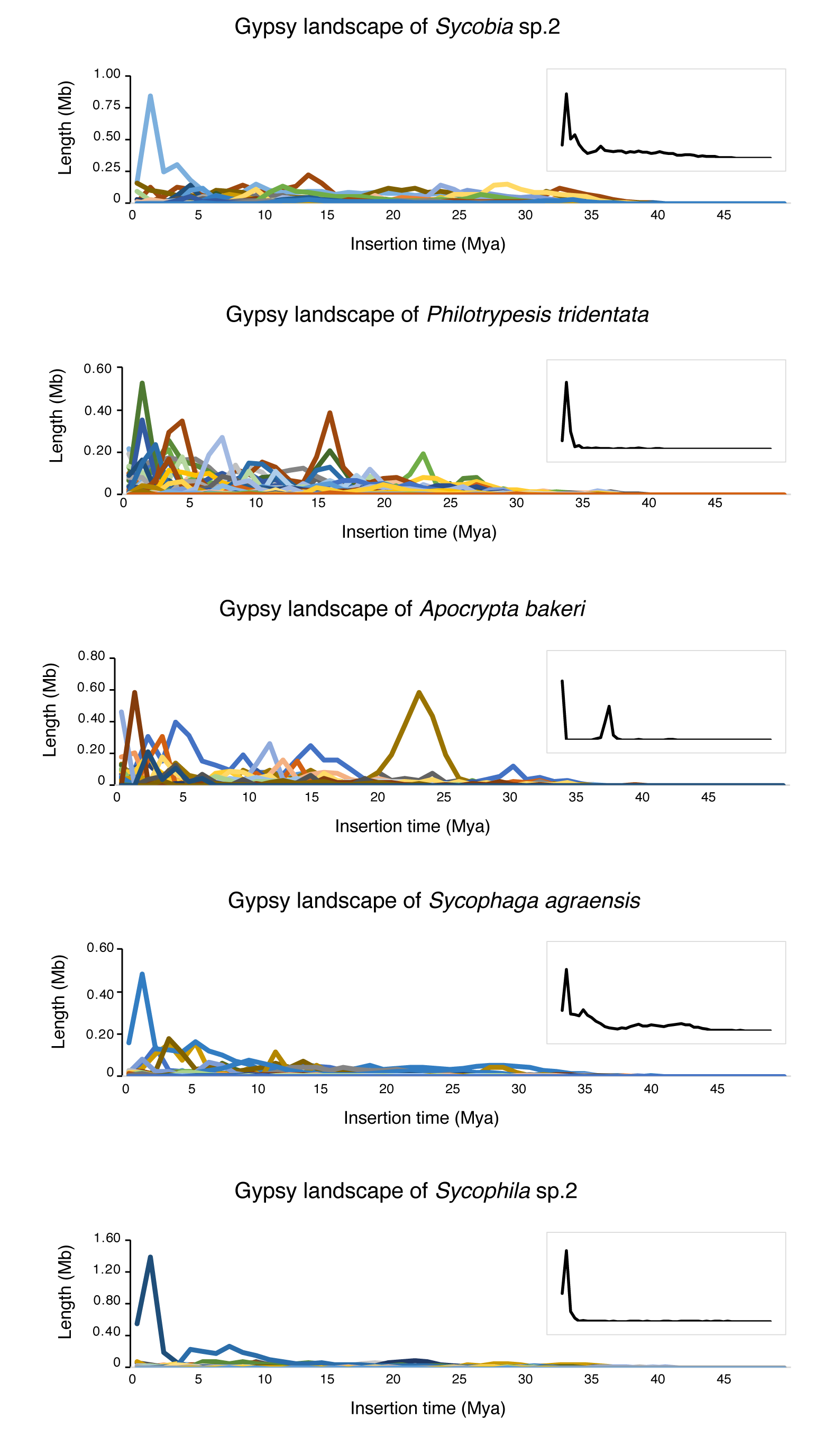

Supplement: Supplementary file 1 — Figure S1. Mann–Whitney U Test Results for TE and Genome Metrics in Fig Wasps. The figure presents the statistical comparisons of transposable element (TE) metrics—including TE length (a), TE count (b), TE type (c), and TE content (d)—as well as genome size (e) between fig wasp groups. These comparisons highlight significant differences in genomic TE characteristics between pollinating and non‐pollinating fig wasps. Figure S2. Gypsy landscape in non‐pollinating fig wasp species. This figure illustrates the distribution of Gypsy transposable elements across various non‐pollinating fig wasp species. The outer panels depict Gypsy insertion patterns and recent bursts in each species, highlighting the frequency and timing of new insertions. The inner panels focus on individual Gypsy elements, revealing multiple burst events over time and showcasing the ongoing and sustained activity of these elements within the genomes. Figure S3. Selection analysis of single‐copy orthologous genes in fig wasps. This figure compares the dN/dS ratios of all single‐copy orthologous genes between pollinating fig wasps (pollinators) and non‐pollinating fig wasps (NPFWs). The median dN/dS ratio is significantly higher in pollinators than in NPFWs, indicating a difference in selective pressure between the two groups (ANOVA, p‐value < 0.001). Figure S4. GO and KEGG network enrichment analysis of genes near classified TEs at the major peak in pollinators. This figure presents the Gene Ontology (GO) and Kyoto Encyclopedia of Genes and Genomes (KEGG) enrichment analysis for genes located near transposable elements (TEs) at the major insertion peak observed in pollinating fig wasps. Only terms with a p‐value < 0.05 were included, as indicated by the filtered nodes in the network. Table S1. One‐sample t‐test for fig wasps in Ficus benjamina . This table presents the results of a one‐sample t‐test comparing the observed traits (genome size, TEs length, TEs count, and TEs content) of NPFWs (Sycobia sp [file ECE3-15-e71553-s001.zip › FIgure_S2_SuppInfo.tif]

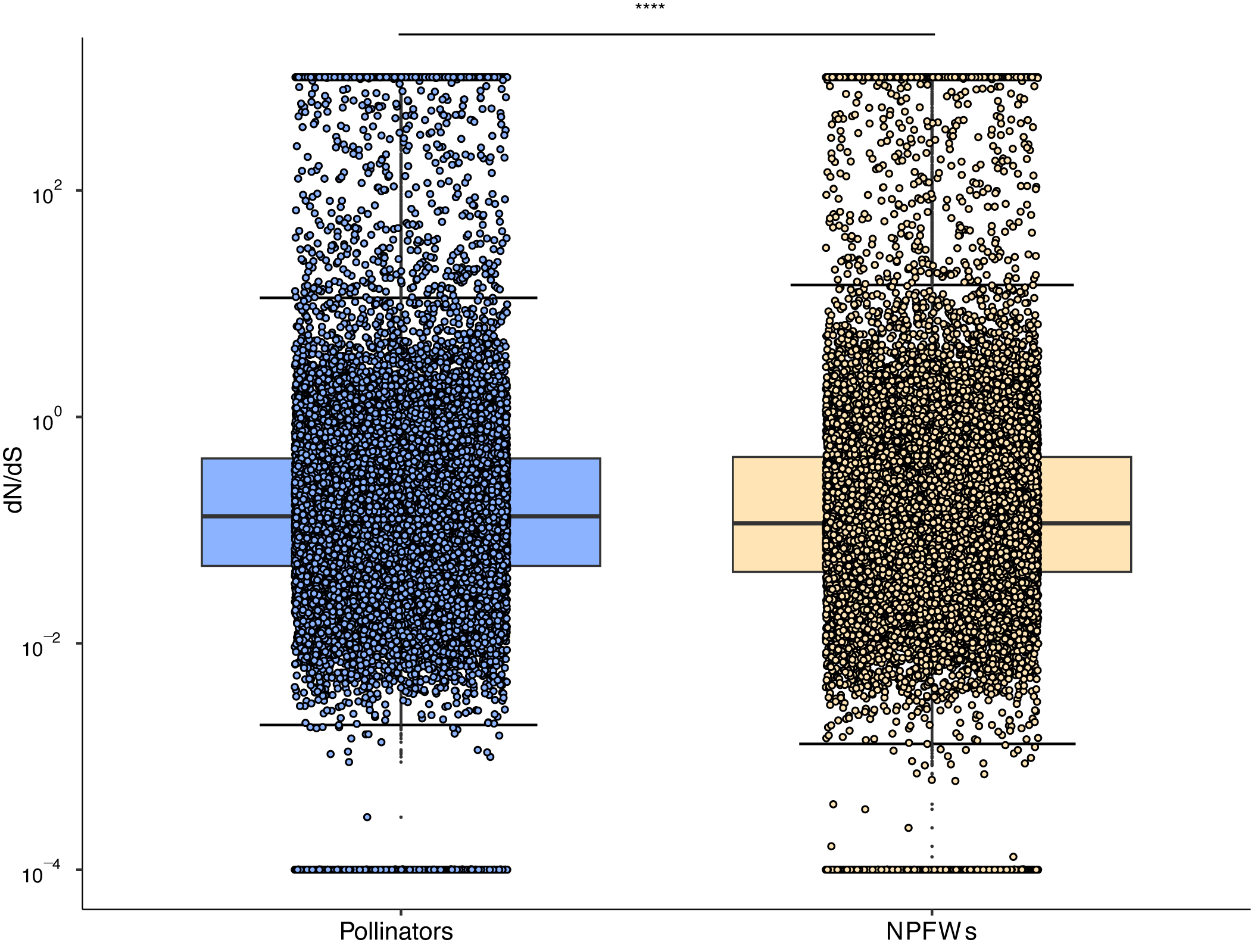

Supplement: Supplementary file 1 — Figure S1. Mann–Whitney U Test Results for TE and Genome Metrics in Fig Wasps. The figure presents the statistical comparisons of transposable element (TE) metrics—including TE length (a), TE count (b), TE type (c), and TE content (d)—as well as genome size (e) between fig wasp groups. These comparisons highlight significant differences in genomic TE characteristics between pollinating and non‐pollinating fig wasps. Figure S2. Gypsy landscape in non‐pollinating fig wasp species. This figure illustrates the distribution of Gypsy transposable elements across various non‐pollinating fig wasp species. The outer panels depict Gypsy insertion patterns and recent bursts in each species, highlighting the frequency and timing of new insertions. The inner panels focus on individual Gypsy elements, revealing multiple burst events over time and showcasing the ongoing and sustained activity of these elements within the genomes. Figure S3. Selection analysis of single‐copy orthologous genes in fig wasps. This figure compares the dN/dS ratios of all single‐copy orthologous genes between pollinating fig wasps (pollinators) and non‐pollinating fig wasps (NPFWs). The median dN/dS ratio is significantly higher in pollinators than in NPFWs, indicating a difference in selective pressure between the two groups (ANOVA, p‐value < 0.001). Figure S4. GO and KEGG network enrichment analysis of genes near classified TEs at the major peak in pollinators. This figure presents the Gene Ontology (GO) and Kyoto Encyclopedia of Genes and Genomes (KEGG) enrichment analysis for genes located near transposable elements (TEs) at the major insertion peak observed in pollinating fig wasps. Only terms with a p‐value < 0.05 were included, as indicated by the filtered nodes in the network. Table S1. One‐sample t‐test for fig wasps in Ficus benjamina . This table presents the results of a one‐sample t‐test comparing the observed traits (genome size, TEs length, TEs count, and TEs content) of NPFWs (Sycobia sp [file ECE3-15-e71553-s001.zip › Figure_S3_SuppInfo.tif]

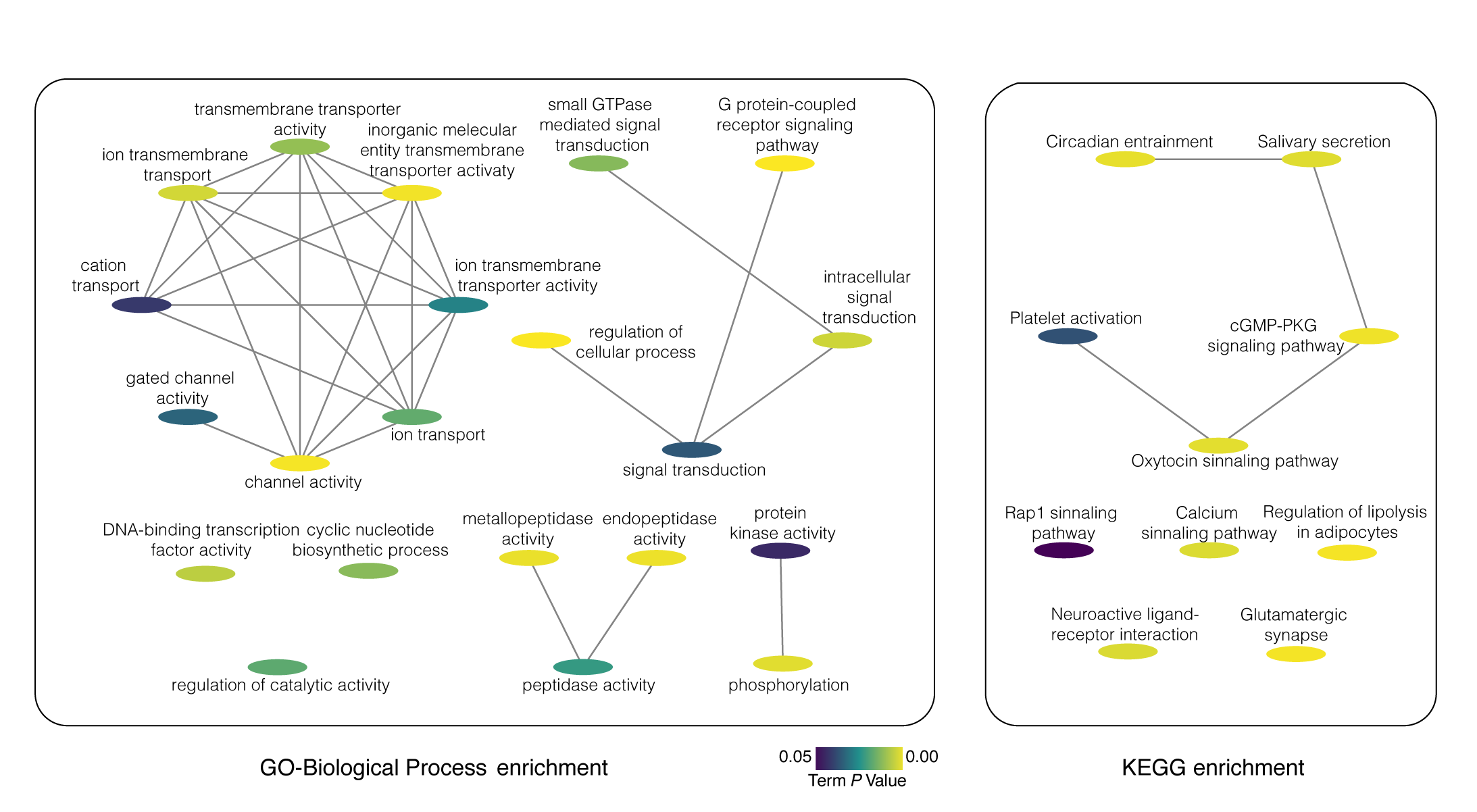

Supplement: Supplementary file 1 — Figure S1. Mann–Whitney U Test Results for TE and Genome Metrics in Fig Wasps. The figure presents the statistical comparisons of transposable element (TE) metrics—including TE length (a), TE count (b), TE type (c), and TE content (d)—as well as genome size (e) between fig wasp groups. These comparisons highlight significant differences in genomic TE characteristics between pollinating and non‐pollinating fig wasps. Figure S2. Gypsy landscape in non‐pollinating fig wasp species. This figure illustrates the distribution of Gypsy transposable elements across various non‐pollinating fig wasp species. The outer panels depict Gypsy insertion patterns and recent bursts in each species, highlighting the frequency and timing of new insertions. The inner panels focus on individual Gypsy elements, revealing multiple burst events over time and showcasing the ongoing and sustained activity of these elements within the genomes. Figure S3. Selection analysis of single‐copy orthologous genes in fig wasps. This figure compares the dN/dS ratios of all single‐copy orthologous genes between pollinating fig wasps (pollinators) and non‐pollinating fig wasps (NPFWs). The median dN/dS ratio is significantly higher in pollinators than in NPFWs, indicating a difference in selective pressure between the two groups (ANOVA, p‐value < 0.001). Figure S4. GO and KEGG network enrichment analysis of genes near classified TEs at the major peak in pollinators. This figure presents the Gene Ontology (GO) and Kyoto Encyclopedia of Genes and Genomes (KEGG) enrichment analysis for genes located near transposable elements (TEs) at the major insertion peak observed in pollinating fig wasps. Only terms with a p‐value < 0.05 were included, as indicated by the filtered nodes in the network. Table S1. One‐sample t‐test for fig wasps in Ficus benjamina . This table presents the results of a one‐sample t‐test comparing the observed traits (genome size, TEs length, TEs count, and TEs content) of NPFWs (Sycobia sp [file ECE3-15-e71553-s001.zip › Figure_S4_SuppInfo.tif]
